# Clinical Characteristics of Non-Melanoma Skin Cancers Recurring within 5 years after Mohs Micrographic Surgery: Single Institution Retrospective Chart Review

**DOI:** 10.13188/2373-1044.1000036

**Published:** 2017-01-10

**Authors:** Tina Vajdi, Robert Eilers, Shang I Brian Jiang

**Affiliations:** 1 Department of Dermatology, School of Medicine, University of California , San Diego, CA, USA; 2 Department of Dermatology, Dermatologic and Mohs Micrographic Surgery Center, School of Medicine, University of California San Diego, CA, USA

## Abstract

**Background:**

Mohs micrographic surgery (MMS) is used to treat certain high-risk non-melanoma skin cancers (NMSC) due to its high cure rate. However, clinical recurrences do occur in a small number of cases.

**Objective:**

We examined specific clinical characteristics associated with NMSC recurrences following MMS.

**Methods:**

We employed a retrospective chart review of the 1467 cases of NMSC that underwent MMS at UC San Diego from January 1, 2008 through December 31, 2009. A total of 356 cases were excluded due to lack of follow-up.

**Results:**

Five (0.45%) of 1111 cases developed recurrences of NMSC at the site of MMS. There were 741 cases of basal cell carcinomas (BCC); 3 were recurrences (0.40%). There were 366 cases of squamous cell carcinomas (SCC); 2 were recurrences (0.55%). Review of MMS histopathology of these recurrent tumors showed that there were no errors or difficulty with the processing or interpretation of the slides.

**Conclusion:**

Five-year recurrence rate of NMSC following MMS at our institution is below the reported average. Our retrospective chart review identified specific clinical characteristics associated with NMSC recurrence including a history of smoking, anatomical location on the cheeks, ears or nose, and a history of immunosuppression for SCCs.

## Introduction

Over one million new cases of non-melanoma skin cancers (NMSC) are diagnosed annually in the United States, and the prevalence of skin cancer is five times higher than that of breast or prostate cancer [1,2]. The incidence of NMSC is increasing worldwide, especially in the United States, Europe and Australia, most likely due to a growing, aging population as well as prolonged ultra violate exposure [3,4].

NMSC most commonly include basal cell carcinomas (BCC) and squamous cell carcinomas (SSC), and many factors influence their development. NMSC such as BCC and SCC most frequently occur on the face and neck due to longitudinal UV exposure [5]. Additionally, patients who are immunosuppressed and/or are organ transplant recipients have significantly increased risk of developing NMSC. There is a 65 to 250-fold increase in developing squamous cell carcinoma and a 10-fold increase in basal cell carcinoma in these patients. In addition to developing higher numbers of NMSC, recent studies have shown that many immunosuppressed patients have NMSCs with aggressive subclinical extensions [6,7]. Associations have also been found between smoking and squamous cell carcinomas, with a higher risk in current smokers than former smokers [8].

The gold standard for the removal of a high risk NMSC is Mohs micrographic surgery (MMS) because of its low recurrence rate and tissue sparing technique. Although there are a number of treatment modalities for NMSC including surgical excision and electrodessication and curettage, MMS continues to be the most advantageous. Studies have shown cure rates for surgical excision as 95% for NMSC less than 2 cm and 84% for electrodessication and curettage for NMSC greater than 2 cm [9]. Unlike other modalities, MMS offers a superior cure rate of 99% within 5 years for primary BCCs, and slightly lower cure rate for primary SCCs [10]. Further analysis has shown the cost effectiveness of MMS in treating high risk NMSC since very high cure rates limit subsequent procedures while maximizing tissue preservation and cosmesis [10,11].

Recurrence rates following MMS have been variable as multiple studies express different recurrence rates. Primary BCCs have about a 1% risk of recurring [10-13]. However, recurrent BCCs have a higher recurrence rate of 10.4% following MMS [6]. Primary SCCs have a recurrence rate of about 2 to 3%, while recurrent SCCs have a 6-10% recurrence rate following MMS [14,15].

In this study, we aim to identify risk factors that lead to NMSC recurrences.

## Methods

We reviewed the medical records of 1467 cases of MMS for NMSC performed at the University of California, San Diego (UCSD) Dermatologic and Mohs Micrographic Surgery Center from January 1^st^, 2008 through December 31^st^ 2009. A retrospective chart review was used to identify cases of NMSC recurrence within 5 years of MMS at our institution. In this IRB approved study, there were 611 cases in which patients lacked 5 years of follow-up at UCSD post MMS. They were contacted via telephone and asked if they had further treatments at the site of the original MMS. Of the 611 cases not followed-up at UCSD, 346 were unable to be reached over the phone (loss to follow-up, new phone numbers, or death) and were excluded from the study. This included 128 SCCs, 217 BCCs, and 1 atypical fibroxanthoma. Three patients who answered the phone were unable to say with certainty if they had further treatments or not on the same location of the initial MMS, and were also excluded from the study. Seven patients had incomplete information in their charts to determine if they had a recurrence in their NMSC, and these cases were excluded. Therefore, 356 cases were excluded from the study.

Retrospective chart review of the 1111 cases included in the study was performed and further subdivided into 741 BCC, 366 SCC and 4 other unusual cutaneous tumors (dermatofibrosarcoma protuberans, porocarcinoma, atypical fibroxanthoma, and sebaceous carcinoma). Recurrent cases were defined by the same type of NMSC, occurring at the same site as the previous MMS. For the recurrent cases, we gathered information regarding the patients’ NMSC histopathology, smoking status, immunosuppression, anatomical location and time to recurrence. Slides from the initial MMS were also reviewed to ensure that there was no error or difficulty with slide interpretation or processing of the slides that would lead to the recurrence.

## Results

Five (0.45%) cases of recurrent NMSC within 5 years were found in the 1111 cases of MMS performed in 2008 and 2009. All five patients underwent MMS within a few months of their NMSC diagnosis. The time to recurrence ranged from 11 to 38 months post initial MMS with an average of 23.6 months. A summary of the data collected can be found in Table 1.

### Demographics of patients with recurrences

All 5 patients (3 male and 2 female) with recurrences were between the ages of 60 and 96. The average age for the initial MMS in these patients was 73 and the average age of MMS for their recurrence was 74.6.

### Histopathology of NMSC-BCC, SCC and other subtypes

There were 741 cases of BBC, 366 cases of SCC and 4 cases of other unusual types of NMSC. There were no recurrences found in the other usual cutaneous tumors including dermatofibrosarcoma protuberans, atypical fibroxanthoma, porocarcinoma and sebaceous carcinoma. There were 3 recurrences of BCC (0.40%) and 2 recurrences of SCC (0.55%).

Two of the three BCC recurrences arose from low-risk nodular BCCs. One of the two SCC recurrences arose from a SCC in situ while the other arose from a biopsy-proven SCC in situ, which was upstaged to a SCC at the time of initial MMS.

The initial MMS histopathology of the recurrent cases was reviewed and no errors or difficulty were noted with the processing or interpretation of the slides.

### Immune status

Both of the SCC recurrences occurred in immunosuppressed patients (one patient with history of lung transplant and another with history of chronic lymphocytic leukemia). The three patients with BCC recurrences were not immunosuppressed.

### Anatomic distribution of recurrences

**Figure 1:**
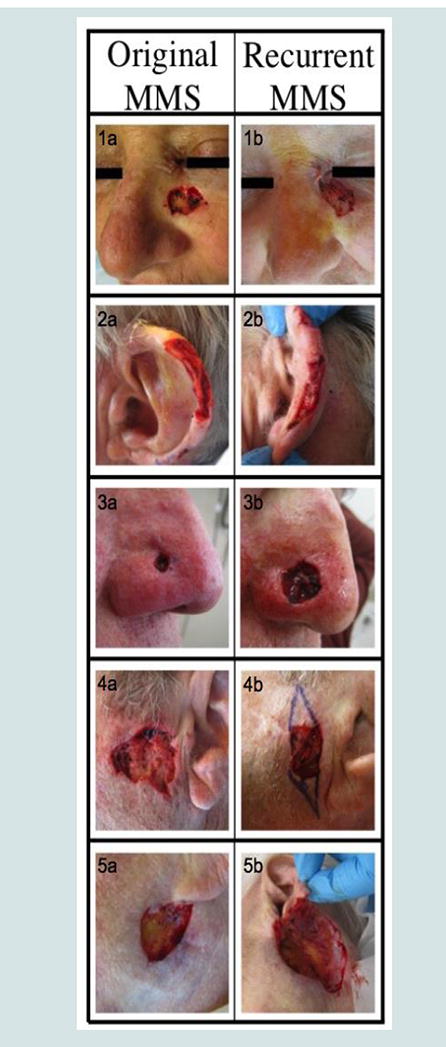
Anatomical location of recurrent NMSC. 1a & 1b: SSC recurrence on left cheek. **2a & 2b**: SCC recurrence on left ear. **3a & 3b**: BCC recurrence on right nasal ala. **4a & 4b**: BCC recurrence on left preauricular area. **5a & 5b**: BCC recurrence on left mandibular area.

All recurrent cases of NMSC were located on the face. Four of the five recurrences involved the left side of patients’ faces, specifically, the left medial cheek, left auricular helix, left preauricular cheek area, and left mandibular cheek. The final recurrence occurred on the right nasal ala (Figure 1).

### Smoking status

Three patients with recurrences also had a history of cigarette smoking. They smoked 0.75 packs per day for 17 years, 1 pack a day for 4 years, and 0.75 packs a day for 15 years.

### Mohs stages and final surgical margins

The number of stages taken for MMS ranged from 1 to 5 (mean 2.6 stages) initially in patients with recurrences, and from 2 to 4 stages (mean 2.6 stages) for their recurrence. Their final postoperative Mohs surgical margins from the longer axis ranged from 2 to 21 mm (mean 12.2 mm) for the initial NMSC, and from 4 to 56 mm (mean 17.8 mm) for the recurrences. Aggressive subclinical extensions (ASE), defined as greater than 3 MMS stages and greater than 1.0 cm final surgical margins, were found in two patients with SCC recurrences and two patients with BCC recurrences as shown in Table 1. Two of the patients had ASE in their initial surgery and two had them in their recurrent surgery.

## Discussion

Our single institution retrospective study revealed a lower than historical recurrence rate in non-melanoma skin cancers after Mohs micrographic surgery. Only 5 (0.45%) of the 1111 cases of MMS performed in 2008 and 2009 had a recurrence within 5 years. Further follow up with these five cases showed no evidence of another recurrence following the second MMS. The range of further follow up for these patients was 12 to 67 months post-MMS for the recurrence (mean 47.4 months). Review of MMS histopathology of these recurrent tumors showed that there were no errors or difficulty with the processing or interpretation of the slides.

There are reports of histologic subtypes being a risk factor for recurrence [16,17]. Our study did not show a correlation between aggressive histopathologic subtypes of NMSC and recurrence.

Immunosuppressed status, including history of solid organ transplant and hematologic malignancies, has been linked to SCC development and aggressive subclinical extensions [18,19] due to their chronic immunosuppressed status and decreased cutaneous immunosurveillance [6,7]. The two patients with SCC recurrences in our study were immunosuppressed (lung transplant and chronic lymphocytic leukemia).

We identified specific clinical characteristics associated with NMSC. All five of the recurrent cases of NMSC were localized to the face, specifically the upper cheek, ear, pre-auricular area and nose (Figure 1), with the final case located partially in the H zone and extending to the cheek. These findings are consistent with other reports in the literature of recurrences following MMS occurring most often in the H zone of the face [20].

A history of smoking was found in 60% of our recurrent cases. There have been several studies showing positive associations between cigarette smoking and development of NMSCs, especially SCC development [21-23]. However, other studies failed to show positive associations between smoking and NMSC development [24,25]. Cigarette smoking as a risk factor for recurrent NMSC has not been studied, and we have found an association between smoking and recurrent NMSC in our study.

Four out of five of the NMSC recurrences were found to have aggressive subclinical extensions (ASE) during the first or recurrent surgery. Both patients with SCC recurrences had ASE and two of the three BCC recurrences had ASE. It is known that immunosuppressed patients, especially those with solid organ transplants are more likely to have NMSC with ASE [7]. This is in accordance with our data as both patients with SCC had ASE and were immunocompromised.

A limitation of this study is that it is a single institution retrospective study that analyzed data of only a two-year period. Since recurrences are rare, further longitudinal and multi-center studies are also warranted to further identity risk factors for NMSC recurrences after MMS.

## Figures and Tables

**Table 1:** Summary of results for patients with recurrent NMSC.

Date of Initial MMS	Date of Recurrent MMS	Location	Smoking Status	Initial Histopathology	Recurrent Histopathology	Immuno-suppression
7/7/09	1/20/11	L cheek	Never smoked	SCC in situ	SCC in situ*	Lung transplant
12/10/09	2/8/11	Left ear (helix)	Formerly	SCC in situ*	SCC	CLL
4/21/09	3/21/12	R nasal ala	Formerly	Nodular BCC	Nodular BCC	
12/1/09	11/9/10	Left preauricular area	Formerly	BCC*	BCC	
8/12/09	10/4/12	Left mandibular area	Never smoked	Nodular BCC	Nodular BCC*	

* NMSC with aggressive subclinical extensions
